# A Data Compression Method for Wellbore Stability Monitoring Based on Deep Autoencoder

**DOI:** 10.3390/s24124006

**Published:** 2024-06-20

**Authors:** Shan Song, Xiaoyong Zhao, Zhengbing Zhang, Mingzhang Luo

**Affiliations:** 1 School of Electronic Information and Electrical Engineering, Yangtze University, Jingzhou 434023, China; songs_yzu@163.com (S.S.); lmz@yangtzeu.edu.cn (M.L.); 2 Directional Drilling Branch of China National Petroleum Corporation Bohai Drilling Engineering Co., Ltd., Tianjin 300280, China; xiaoyong2810@163.com

**Keywords:** wellbore safety monitoring, deep autoencoder, well trajectory, data compression, logging while drilling (LWD)

## Abstract

The compression method for wellbore trajectory data is crucial for monitoring wellbore stability. However, classical methods like methods based on Huffman coding, compressed sensing, and Differential Pulse Code Modulation (DPCM) suffer from low real-time performance, low compression ratios, and large errors between the reconstructed data and the source data. To address these issues, a new compression method is proposed, leveraging a deep autoencoder for the first time to significantly improve the compression ratio. Additionally, the method reduces error by compressing and transmitting residual data from the feature extraction process using quantization coding and Huffman coding. Furthermore, a mean filter based on the optimal standard deviation threshold is applied to further minimize error. Experimental results show that the proposed method achieves an average compression ratio of 4.05 for inclination and azimuth data; compared to the DPCM method, it is improved by 118.54%. Meanwhile, the average mean square error of the proposed method is 76.88, which is decreased by 82.46% when compared to the DPCM method. Ablation studies confirm the effectiveness of the proposed improvements. These findings highlight the efficacy of the proposed method in enhancing wellbore stability monitoring performance.

## 1. Introduction

Wellbore instability is a significant challenge encountered during drilling operations in diverse oil and gas reservoirs [1,2]. It encompasses issues such as collapse, shrinkage, diameter enlargement, and fracture, all of which can impede drilling efficiency and, if left unchecked, result in serious incidents. Therefore, it is imperative to monitor wellbore stability to ensure safe drilling practices.

At present, the primary method for monitoring wellbore health [1,3] involves the use of logging while drilling (LWD) tools to gather real-time data on the wellbore’s condition. The data are then evaluated using established rock mechanics principles and correlations with logging data. However, this approach is limited by the low data transmission rates of LWD. The most common data transmission technology used in LWD is mud pulse telemetry (MPT), which operates at a rate of 0.5 bit/s to 1.0 bit/s [4,5,6]. Consequently, it is challenging to meet the real-time logging engineering requirements with this transmission rate. While stress wave-based communication using piezoceramic transducers offers higher data transmission rates [7,8,9], this technology is still in the laboratory research stage and has not yet been commercialized [10].

Given the current constraints of MPT or stress wave-based communication bandwidth, transmitting compressed data for wellbore stability monitoring can significantly enhance real-time performance. This improvement is crucial for maintaining wellbore safety and stability while enhancing drilling efficiency. Among the parameters vital for wellbore stability monitoring, inclination, azimuth, and depth are particularly critical [11]. During drilling operations, continuous monitoring and adjustment are essential to ensure the wellbore trajectory aligns optimally, especially in formations with narrow safety margins [12]. Consequently, researching compression methods for inclination and azimuth data becomes imperative.

Currently, the predominant compression techniques for logging while drilling (LWD) data include the methods based on Huffman coding [13], compressed sensing [14], wavelet transform [15], long short-term memory (LSTM) [16], and the Differential Pulse Code Modulation (DPCM) method [17]. Huffman coding, a classic lossless compression method, allows for complete restoration of original data from the compressed data, albeit with relatively low compression rates. For instance, Song et al. [18] employed Frame Prediction Huffman Coding (FPHC) in their 2021 study to compress various logging data, achieving a compression ratio of only 1.41. Although this ratio surpasses that of traditional Huffman and arithmetic coding, its impact on enhancing data transmission efficiency remains limited. The compressed sensing method utilizes the sparsity of logging while drilling data to collect logging data at a rate much lower than Nyquist sampling, thereby reducing the amount of data collected and achieving the effect of data compression. For instance, in 2020, Li et al. [19] proposed a compressed sensing method based on dictionary machine learning, which constructs and adjusts a dictionary through machine learning to compress the downhole density data. Compared with traditional averaging and interpolation methods, this method has a compression ratio of 3.33 and a mean square error 18 times smaller than traditional methods. However, this method requires obtaining enough sampling data (128 sampling data) before compression and transmission can begin, resulting in low real-time performance. Wavelet transform represents a signal as a scaled and shifted “mother wavelet” oscillation waveform. After matching the signal, only the corresponding parameters need to be transmitted to effectively represent the original signal, thus achieving effective compression of the signal. For instance, Jarrot et al. [20] proposed the method of directional wavelet transform to compress downhole image data in 2018. The results showed that for images with a width of 80 pixels and a height of 256 pixels, the rate reached 0.1 bits/pixel (with a compression ratio of 80), and the tilt angle could still be clearly identified. However, this method requires obtaining two-dimensional data with a width of 80 pixels and a height of 256 pixels for compression. Even if each pixel occupies an average of 0.1 bits, when transmitting compressed data, it still needs to transmit 2048 bits to express a downhole image. As a result, for the currently widely used MPT system, the waiting time for transmission would be very long. The LSTM method achieves data compression mainly by predicting the signal and then compressing and encoding the difference between the original data and the predicted data. Yan Zhidan et al. [21] used the LSTM and XGBoost method [22] to predict the main and detailed data of LWD after discrete wavelet transform, and then quantified and encoded the difference between the original data and the predicted data. The compression ratio reached two, and the distortion rate was only 0.01%. However, this method uses wavelet transform and the LSTM network, which requires a long time to obtain sufficient data volume to achieve ideal prediction results and a sufficient compression ratio. Therefore, the real-time performance of this method is also poor. The DPCM method is easy to implement, with no requirement for the amount of collected data; thus, it has good real-time performance. For instance, Zhang et al. [23] proposed in 2009 to use the DPCM method to compress gamma and resistivity data. The predictor used was a first-order predictor, while the quantizer used three-bit and four-bit quantization bits, respectively. The results showed that the compression ratio of the DPCM could reach more than two, and the distortion remained within 5%. The following year, Zhang et al. conducted further research on the DPCM method [24]. They used multiple predictor orders and multiple quantization bits to compress various logging data, such as gamma, resistivity, and temperature, for a wider range of logging parameters, achieving a compression ratio of two and a distortion rate below 5%. Despite its superior compression ratios compared to Huffman coding and good real-time performance, the DPCM method still falls short in practical engineering applications due to its relatively low compression efficiency. Additionally, abrupt data changes often result in significant decoding errors with the DPCM method [17,24]. Given the commonplace occurrence of rapidly changing LWD data in practice, these decoding errors render the DPCM method unreliable. Among the above methods, the compression ratio of Huffman coding-based compression methods is very low and difficult to improve. Although compression sensing, wavelet transform, and LSTM-based compression methods have higher compression ratios, these methods require a large amount of collected data, often requiring hundreds or thousands of data points to achieve effective compression. In M/LWD engineering, the sampling interval of inclination, azimuth, gamma, and other logging data is usually in seconds. If these methods are used, it will take a long time to complete the compression and transmission of data, which will inevitably cause serious information lag. Relatively, the DPCM method can achieve real-time transmission of logging data, and its compression ratio is higher than the lossless compression method based on Huffman coding. However, the compression ratio of this method is still relatively low and the error is large, making it difficult to meet the needs of real-time monitoring of wellbore stability. Addressing the limitations of existing compression methods, it becomes imperative to develop novel compression methods tailored to wellbore trajectory data.

In recent years, machine learning methods have emerged as potent tools for signal processing [25,26,27]. Among these, the autoencoder stands out as an unsupervised artificial neural network renowned for efficiently learning data features, often utilized for data dimensionality reduction [28,29]. Comprising two main components—the encoder and the decoder—the autoencoder operates by first reducing the dimensionality of the data, thereby reducing the volume of data and facilitating data compression. Subsequently, the decoder reconstructs the data to closely resemble the input data [30,31]. Leveraging its capacity for dimensionality reduction and reconstruction, autoencoders find widespread application in data compression. For instance, Nuha et al. [32] introduced a stacked autoencoder extreme learning machine (AE-ELM) for seismic data compression. Achieving a compression ratio of 10, the normalized mean square error (NMSE) between the reconstructed seismic data and the original data was recorded at 1.28 × 10^−3^, surpassing the performance of the classic Discrete Cosine Transform (DCT) method. This underscores the efficacy of autoencoder-based approaches in seismic data compression. Similarly, in 2021, Liu et al. [33] conducted a pioneering compression study on HPC (high-performance computing) scientific data using an autoencoder. Notably, the compression ratio of the HACC dataset in the scientific data reduction benchmark dataset reached 241.67, marking a four-fold increase compared to the classic Squeeze (SZ) [34] compression method and a fifty-fold increase over the ZFP [35] method. This study underscores the compelling compression capabilities of autoencoders for HPC scientific data. Moreover, autoencoders have found substantial applications in image compression for both research and practical purposes [36,37,38].

Compared to classical autoencoders, the deep autoencoder proposed by Hinton et al. [39] demonstrates superior performance in learning data features and reducing data dimensionality. Structurally akin to autoencoders, deep autoencoders comprise an encoder and decoder, yet each component boasts multiple neural network layers. This architecture allows for more effective learning of data feature representations and dimensionality reduction, making these autoencoders promising candidates for data compression across various domains. For instance, Yildirim et al. [40] employed deep convolutional autoencoders to compress electrocardiogram (ECG) signals, achieving a compression ratio of 32.25 with a percentage RMS difference (PRD) of 2.73%. This outperforms classic methods such as DWT + RLE (Discrete Wavelet Transform + Run Length Encoding) and DCT + RLE + Huffman. Similarly, Kuester et al. [41] utilized a deep autoencoder to compress a representative set of spectral data in 2020, achieving a compression ratio of four with an almost lossless compression process.

The dynamic ranges of inclination and azimuth data in wellbore trajectory parameters are relatively large, typically requiring 11 bits for encoding. Consequently, traditional compression methods like Huffman coding and DPCM are not suitable for this task. Deep autoencoders, renowned for their adeptness at learning data features, offer a promising alternative. By leveraging their capability to extract key features from high-dimensional data and obtain low-dimensional representations, deep autoencoders can effectively compress inclination and azimuth data. In light of these considerations, we propose a data compression method based on deep autoencoders specifically tailored for compressing inclination and azimuth data. This approach aims to enhance the compression ratio, addressing the issue of low compression ratios encountered with existing methods.

Our proposed method involves subtracting the reconstruction data of the autoencoder from the original data to obtain residual data, which is then compressed. As the residual data typically exhibits low correlation characteristics, quantization coding is applied for compression, complementing the compressed data from the autoencoder. This integrated approach effectively reduces the error between the reconstruction data and the source data. Furthermore, mean filtering based on a standard deviation threshold is employed to further mitigate errors between the reconstructed data at the decoding end and the source data. However, it is essential to note that not all data in the reconstructed data are suitable for mean filtering. Data with significant changes, indicated by large standard deviations, may actually incur increased errors when subjected to mean filtering. To address this, we propose utilizing a standard deviation threshold to control the mean filter, filtering only the data below this threshold. To optimize the selection of the standard deviation threshold and minimize reconstruction data errors, we employ the Root Mean Square Propagation (RMSProp) [42] optimization algorithm. This approach ensures efficient parameter tuning, thereby enhancing the overall performance of the compression method. The contributions of this paper are summarized as follows:

- We propose an efficient and real-time compression method for wellbore safety monitoring-related data, which effectively improves the compression efficiency of wellbore inclination and azimuth data while greatly reducing the error between the reconstructed data and the original data. This solves the problems of low real-time performance, low compression efficiency, and large reconstruction data error in existing methods, and can effectively improve the performance of wellbore stability monitoring;
- We propose for the first time the use of deep autoencoders to compress inclination and azimuth data, achieving significant compression of inclination and azimuth data, effectively solving the problem of low compression ratio in existing methods;
- We propose a mean filtering method based on the optimal standard deviation threshold to filter the reconstructed data after compensation of the residual, further effectively reducing the error between it and the original data.

The rest of the paper is organized as follows: Section 2 introduces the basic principles and provides a detailed process of the proposed method. Section 3 introduces the experimental data and experimental setup. Section 4 showcases the results of the simulation experiments. Finally, Section 5 offers concluding remarks to summarize the key findings and implications of the study.

## 2. Proposed Method

### 2.1. The Overall Framework of the Proposed Method

#### 2.1.1. Block Diagram of Compressed Data Transmission System for LWD

**Figure 1:**
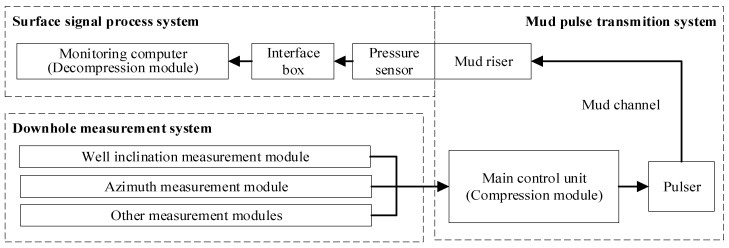
Block diagram of compressed data transmission system for LWD.

In order to improve the transmission efficiency of the data about wellbore stability monitoring and enhance real-time monitoring, it is necessary to embed the compression method into the MPT system, forming a compressed data transmission system for LWD. The structural diagram of the compressed data transmission system for LWD is shown in Figure 1, which is mainly divided into downhole measurement systems, mud pulse transmission systems, and surface signal processing systems.

The downhole measurement system comprises various modules for measuring parameters like inclination, azimuth, and other logging parameters. In particular, the inclination and azimuth measurement modules acquire data regarding the downhole equipment’s inclination and azimuth angles, which need to be compressed and transmitted to improve the performance of monitoring wellbore stability.

The mud pulse transmission system is composed of the main control unit, pulser, mud channel and mud riser. Embedded within the main control unit, the data compression module—whether in software or hardware form—compresses and encodes inclination and azimuth data. This compression reduces their code length, thereby enhancing transmission efficiency and ultimately improving wellbore stability monitoring performance. During operation, the main control unit acquires data from the downhole measurement system via the bus. Subsequently, it compresses and encodes the inclination and azimuth data, integrating them with other measurement data. After encoding and packaging, the pulser emits pulses to alter the circulating mud pressure within the drill string in the mud channel, transmitting the signal to the surface as mud pressure waves. Finally, the signal reaches the surface signal processing system through a mud riser.

The surface signal processing system comprises pressure sensors, interface boxes, and a monitoring computer. Within the monitoring computer, the decompression module is integrated into the signal processing software. This module decompresses the compressed inclination and azimuth data, generating their reconstruction data, which reflects the inclination and azimuth information of the downhole equipment. During operation, the pressure sensors detect changes in mud fluid pressure, generating signals that are transmitted to the monitoring computer via the interface box. Within the monitoring computer, the signal processing software processes these pressure signals, removing noise and decoding them to obtain compressed inclination and azimuth data. Subsequently, through the decompression module, the wellbore inclination and azimuth information are reconstructed and displayed, facilitating real-time monitoring of wellbore stability.

To enhance the monitoring performance of wellbore stability, it is necessary to effectively compress and decompress inclination and azimuth data through compression and decompression modules. However, existing compression methods suffer from low real-time capability, low compression ratios, and significant errors between the reconstructed and raw data. To address these issues and enhance the effectiveness of wellbore stability monitoring, a novel approach to data compression must be proposed.

#### 2.1.2. Structural Diagram of the Proposed Method

To boost the compression ratio of inclination and azimuth data, and considering the efficient data dimensionality reduction ability of deep autoencoders, we leverage the dimensionality reduction capability of deep autoencoders, enabling significant compression of the raw data. However, there is an error between the reconstructed data of the deep autoencoder and the original data. The error is primarily due to the complete discarding of the residual between the reconstructed data and the original data; therefore, to reduce the error, it is necessary to compress and transmit the residual data. To this end, we use quantization coding and Huffman coding to compress the residual data, and then compensate for the reconstructed data of the deep autoencoder. Quantization coding can effectively reduce the error between the reconstructed data of the deep autoencoder and the original data, but it will also lead to a reduction in the compression ratio. To minimize the reduction in the compression ratio, we use Huffman coding to further compress the data encoded by quantization coding. In addition, since quantization coding reduces the dynamic range of the residual data, the amount of codeword information required for the Huffman coding is also reduced, making Huffman coding easier to implement in downhole equipment. The introduction of quantization coding and Huffman coding can effectively reduce the error while still maintaining a high compression ratio.

Additionally, we apply mean filtering based on a standard deviation threshold to the compensated reconstructed data. This step is crucial because even after compensation, the reconstructed data still contains errors compared to the raw data, akin to noise interference. These errors primarily stem from the deep autoencoder’s inability to fully extract all features of the raw data. Hence, mean filtering is necessary to mitigate this interference. However, not all compensated reconstructed data is suitable for filtering. For segments of data that exhibit stability (with a small standard deviation), the error interference is more pronounced, and mean filtering effectively reduces this interference. Conversely, for segments of data undergoing significant changes (with a large standard deviation), applying mean filtering would exacerbate errors rather than mitigate them.

**Figure 2:**
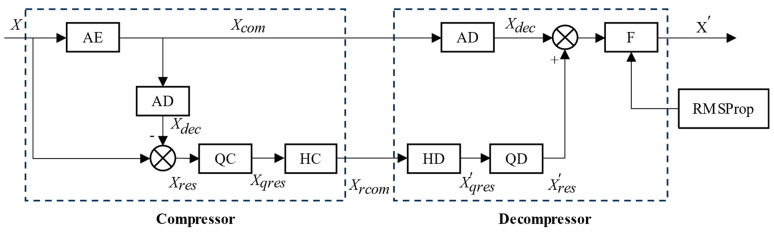
Diagram of data compression method based on deep autoencoder.

We employ a standard deviation threshold as a criterion to determine whether a particular segment of the data requires mean filtering. To establish an optimal standard deviation threshold that minimizes the error between the final reconstructed data and the raw data, we leverage the RMSProp optimization algorithm. RMSProp is chosen for its stability and rapid convergence, making it an ideal candidate for this task. Figure 2 illustrates the compression method based on a deep autoencoder, incorporating the aforementioned concepts.

The compression method based on deep autoencoder proposed in this paper consists of two parts: a compressor and a decompressor. During compression, the original data and residual data are compressed separately. On one hand, the compressor compresses the source data X with the encoder of the deep autoencoder (AE) to obtain compressed data $X_{\mathrm{com}}$, and $X_{\mathrm{com}}$ will be directly transmitted to the decompressor. On the other hand, the compressor decompresses $X_{\mathrm{com}}$ with the decoder of the deep autoencoder (AD) to obtain the decompressed data $X_{\mathrm{dec}}$, then $X_{\mathrm{dec}}$ is subtracted from X to obtain residual data $X_{\mathrm{res}}$. Subsequently, the quantization coding method (QC) is used to encode $X_{\mathrm{res}}$ and obtain $X_{\mathrm{qres}}$, then compress $X_{\mathrm{qres}}$ with Huffman coding (HC) to obtain the compressed data ($X_{\mathrm{rcom}}$) of residual $X_{\mathrm{res}}$ and pass it to the decompressor.

The decompressor is also divided into two parts. The first part uses the decoder of the deep autoencoder (AD) to decompress $X_{\mathrm{com}}$ to obtain the decoded data of the deep autoencoder ($X_{\mathrm{dec}}$). The second part decompresses the compressed residual data $X_{\mathrm{rcom}}$ with the Huffman decoding method (HD) to obtain the quantization encoding data of residual data $X_{\mathrm{qres}}$, then decompresses $X_{\mathrm{qres}}$ with the quantization decoding method to obtain the reconstructed data of residual data $X_{\mathrm{res}}^{\prime }$. Then, $X_{\mathrm{dec}}$ is added to $X_{\mathrm{res}}^{\prime }$, and the output of the adder is filtered with filter F to obtain the reconstructed data $X^{\prime }$. Filter F adopts the mean filtering method based on the optimal standard deviation threshold and should be trained using the RMSProp optimization algorithm with a training dataset measured in advance to obtain the optimal standard deviation threshold.

Below are the principles of deep autoencoder, quantization coding, and Huffman coding, as well as the detailed implementation process of the proposed method.

### 2.2. Extraction of Source Data Features

The proposed method uses a deep autoencoder to extract features from the source data, which can significantly improve the compression ratio. Deep autoencoders are developed based on autoencoders. The following section will introduce the concepts of autoencoder and deep autoencoder.

#### 2.2.1. Autoencoder

**Figure 3:**
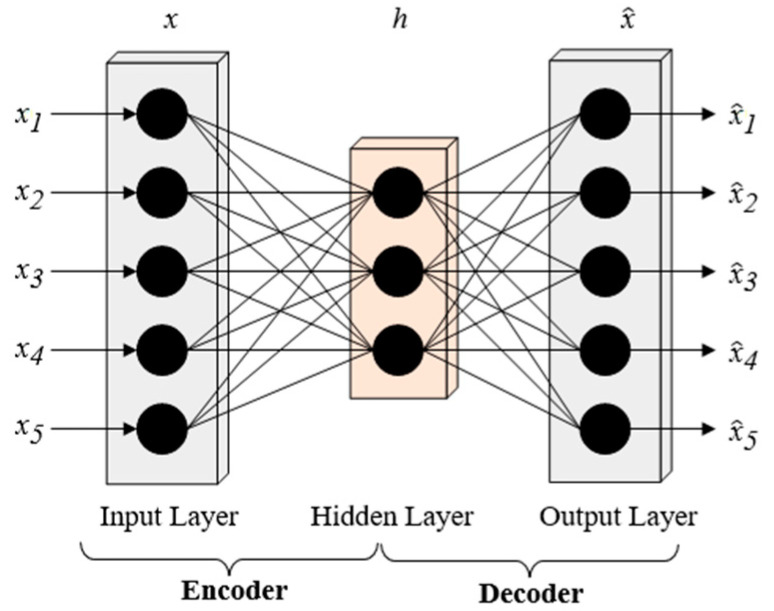
Autoencoder structure.

Autoencoder is an unsupervised neural network with a symmetric structure, which can effectively learn the internal features of data [43] to obtain concise expressions of data; it is often used for data dimensionality reduction [44]. The standard autoencoder has a three-layer architecture [45], as shown in Figure 3. Autoencoder is structurally composed of an input layer, a hidden layer, and an output layer. According to its function, autoencoder can be further divided into an encoder and decoder. The encoder includes an input layer and a hidden layer, responsible for compressing high-dimensional input data $x\in R^{n}$ to obtain its low-dimensional feature representation $h\in R^{\hat{n}}$ ($\hat{n}<n$) to achieve data compression. The decoder includes the hidden layer and an output layer, responsible for reconstructing data x using feature representation h to obtain reconstructed data $\hat{x}\in R^{n}$ and try to ensure that $\hat{x}$, as much as possible, approaches the input raw data x.

The formulas for the encoder and decoder are as follows:

(1) $$h=f\left(W_{h}x+b_{h}\right)$$

(2) $$\hat{x}=f\left(W_{\hat{x}}h+b_{\hat{x}}\right)$$

where x is the input data, and h is the compressed data for feature representation. $\hat{x}$ is the reconstructed data. $W_{h}$ and $b_{h}$ are the weights and bias between the input layer and the hidden layer. $W_{\hat{x}}$ and $b_{\hat{x}}$ are the weights and bias from the hidden layer to the output layer. f(Wx+b) is an activation function, usually a nonlinear function such as a sigmoid function or a hyperbolic tangent function.

The goal of training autoencoder is to find the optimal matrix $W_{h}$, $W_{\hat{x}}$, $b_{h}$, and $b_{\hat{x}}$ to minimize the error between the input data *x* and the reconstructed data $\hat{x}$, as follows:

(3) $$\underset{W_{h},W_{\hat{x}},b_{h},b_{\hat{x}}}{\arg\;\min}\left[d\left(x,\hat{x}\right)\right]$$

where $d(x,\hat{x})$ is a loss function used to characterize error between the input data *x* and the reconstructed data $\hat{x}$. During training, parameters are updated using backpropagation algorithms and optimization algorithms such as adaptive moment estimation (Adam). The backpropagation algorithm is used to calculate the gradient of a multivariate function, while the optimization algorithm updates $W_{h}$, $W_{\hat{x}}$, $b_{h}$, and $b_{\hat{x}}$ based on the direction in which the gradient of the loss function decreases. After the training, the loss function converges to the local or global minimum value, and the generated autoencoder model is used for feature extraction and dimensionality reduction of input data.

#### 2.2.2. Deep Autoencoder

**Figure 4:**
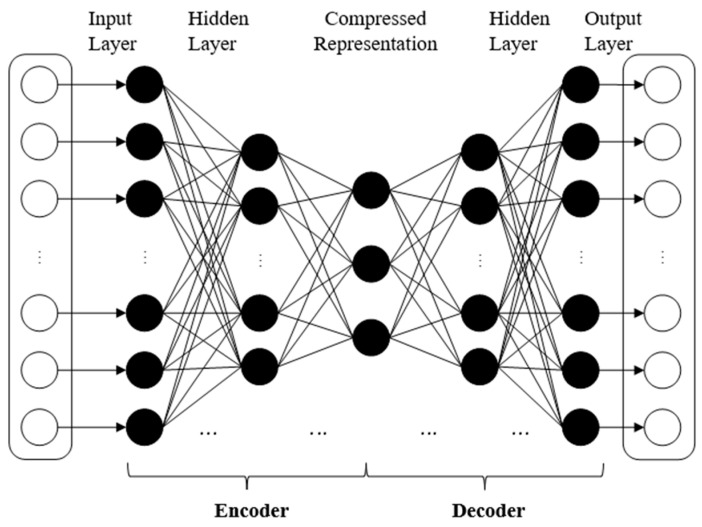
Deep autoencoder structure.

Compared to autoencoder, deep autoencoder has more hidden layers, and the output of each layer constitutes the input of the next layer. As shown in Figure 4, except for the input and output layers, all other layers in the figure are hidden layers, and the hidden layer in the middle has the smallest dimension, which will be used as a compressed representation.

Deep autoencoders have a better ability to learn data features; therefore, we use a deep autoencoder to compress inclination and azimuth data. The deep autoencoder structure information used in the proposed method is listed in Table 1.

All layers are dense layers, among which the encoder includes hidden layer 1, hidden layer 2, and the bottleneck layer. The bottleneck layer has the least number of data nodes, and its output value is the compressed data. The decoder includes hidden layer 3, hidden layer 4, and hidden layer 5. The output data of hidden layer 5 is the decoded data, and its dimension is exactly the same as the input data dimensions of hidden layer 1.

The computation method for the compression ratio of deep autoencoder is as expressed by Equation (4).

(4) $$C_{R}=\frac{nb_{\mathrm{in}}}{\hat{n}b_{\mathrm{BN}}}$$

where n is the dimension of the input data, and $\hat{n}$ is the dimension of the bottleneck layer. $b_{\mathrm{in}}$ is the bit length of the input data, and $b_{\mathrm{BN}}$ is the bit length of the output data of the bottleneck layer (compressed data). The activation function of each hidden layer adopts Leaky_ReLU, and its formula is as follows:

(5) $$\phi =f\left(x\right)=\left\{\begin{array}{c} ax,\;x<0\\ x,\;x\geq 0 \end{array}\right.$$

where *a* > 0. The reason we opt for the Leaky_ReLU function is its capability to prevent gradient vanishing [46] while also introducing nonlinear transformation characteristics to the network.

### 2.3. Compression of Residual Data

After the deep autoencoder compresses the source data, further feature extraction from the remaining residual data becomes challenging. Simply discarding this residual data is not conducive to reducing the error between the reconstructed data and the source data. Hence, we employ quantization coding and Huffman coding to compress the residual data. In the decompressor, this compressed residual data is reconstructed to compensate for the reconstruction data of the deep autoencoder, thereby reducing the error between the reconstructed data and the source data. Quantization coding serves to preliminarily compress the residual data, thereby reducing its dynamic range, while Huffman coding effectively compresses the data.

#### 2.3.1. Quantization Coding

Quantization coding uniformly uses smaller bit lengths $B_{q}$ to represent data that are originally needed to be represented by $B_{in}$ bit lengths ($1<B_{q}<B_{\mathrm{in}})$. The quantization encoding formula is as follows:

(6) $$d_{n}^{\prime }=Q\left(d_{n}\right)=\frac{b_{i-1}+b_{i}}{2}\left(b_{i-1}\leq d_{n}<b_{i}\right)$$

(7) $$b_{j}=-2^{B_{\mathrm{in}}-1}+j\prime \frac{2^{B_{\mathrm{in}}}}{2^{B_{q}}}\left(j=0,\;1,\;2\ldots 2^{B_{q}}\right)$$

where $b_{i}$ is the boundary value of the quantization interval, i is the quantization level, and $i=1,\;2\ldots 2^{B_{q}}$, whose value needs to be determined through traversal. By taking $i=1,\;2\ldots 2^{B_{q}}$ when $b_{i-1}\leq d_{n}<b_{i}$ is established, the value of i is the quantization level corresponding to the current data.

The quantization coding encodes the residual quantization value $d_{n}^{\prime }$ based on the bit length of quantization coding $B_{q}$. Since the total value of $B_{q}$ is less than $B_{d}$, the dynamic range of the required encoded data is reduced, which is beneficial for further compression using Huffman coding in the subsequent step.

#### 2.3.2. Huffman Coding

The principle of Huffman coding is to assign Huffman codes to data based on the frequency or probability distribution of the source data. Among them, data with high frequency or probability are assigned codes with fewer encoding bits, while data with low frequency or probability are assigned codes with more encoding bits. In this way, most of the source data will be encoded into Huffman code with fewer bits, thereby reducing the overall amount of encoded data and achieving compression. Huffman coding is a commonly used and easily implementable lossless data compression method, often serving as part of a more complex data compression method. Huffman coding is a relatively common, efficient, and easily implementable lossless data compression method, often used as part of a more complex data compression method. The detailed principles and implementation methods can be found in ref. [13]. This paper utilizes Huffman coding to compress the data encoded by quantization coding, in order to reduce the impact of the reduced compression ratio caused by quantization coding.

### 2.4. Mean Filtering of Compensated Reconstructed Data

After obtaining the compensated reconstruction data, there still exists a certain degree of error compared to the source data. Filtering can further mitigate this error between the compensated reconstruction data and the source data. Therefore, we employ mean filtering to process the compensated reconstruction data. However, the reconstructed data encompasses various data characteristics, and not all data are suitable for filtering. For data exhibiting significant changes, mean filtering may exacerbate errors, while for data with subtle changes, it can effectively reduce the error between the reconstructed data and the source data. To discern these data characteristics, we utilize standard deviation. Thus, it is essential to determine an appropriate standard deviation threshold. If the standard deviation surrounding the current data is less than this threshold, mean filtering is applied; otherwise, no processing is conducted. The value of the standard deviation threshold is correlated with the error between the reconstructed data and the source data. To select the optimal standard deviation threshold and minimize the error, we leverage the RMSProp optimization algorithm for training.

#### 2.4.1. RMSProp Optimization Algorithm

Obtaining the standard deviation threshold to minimize the reconstruction data error is a type of optimization problem. The RMSProp algorithm is a suitable optimization algorithm, which has good convergence stability since it only solves for the average value of gradients within a certain duration [47]. At the same time, this method has a faster convergence rate by discarding the early gradients. Therefore, we adopt the RMSProp algorithm to obtain the optimal standard deviation threshold as

(8) $$v_{t}=\beta \cdot v_{t-1}+\left(1-\beta \right){\left(g_{t}\right)}^{2}$$

(9) $$w_{t+1\;}=w_{t}-\frac{\mathrm{lr}\cdot g_{t}}{\sqrt{v_{t}+\varepsilon }}$$

where β is a hyper-parameter; the larger its value, the greater the weight of the memorized historical gradient, usually around 0.9. ε is a very small constant, mainly designed to avoid being divided by zero, usually taking a value of $10^{-6}$. lr is the learning rate, which can generally be taken as 0.01. $w_{t}$ is the weight of the *t*-th iteration, which is the value to be solved, while $g_{t}$ is the gradient of the loss function relative to $w_{t}$. Their relationship is

(10) $$g_{t}=\nabla_{w_{t}}f\left(w_{t}\right)$$

where $f\left(w_{t}\right)$ is the loss function. To find the optimal standard deviation threshold and minimize the error between the compensated reconstructed data and the source data, the standard deviation threshold can be used as $w_{t}$, and the mean square error MSE can be used as the loss function. When iterating using the RMSProp formula mentioned above, the value of $w_{t}$ corresponding to the convergence of the loss function value is the optimal value of standard deviation threshold.

#### 2.4.2. Mean Filtering Method Based on Optimal Standard Deviation Threshold

After obtaining the optimal standard deviation threshold, selective filtering is performed on the compensated reconstructed data based on this threshold to further reduce the error between the reconstructed data and the source data. The filtering method used in this paper is the mean filtering, which is a linear filter that takes the average value of the data as the output value of the center point. If the optimal standard deviation threshold obtained through the optimization algorithm is $T_{W}$, the implementation process of the mean filtering method based on the standard deviation threshold $T_{W}$ is as follows:

Step 1: Calculate the index of the center point and the end point data within the filtering window. The calculation method is as follows:

(11) $$i_{e}=i+N_{W}-1$$

(12) $$i_{c}=\frac{2i+N_{W}-1}{2}$$

where $N_{W}$ is the size of the filtering window; its value should not be too large because the larger the value, the more points that are required for filtering, which will delay the time for filtering. This is not favorable for the surface system to quickly obtain the filtered reconstructed data, and it may cause more significant errors at the beginning of the drilling operation. Meanwhile, its value should not be too small, as the filter will not be able to accurately distinguish between noise and collected signals, leading to poor filtering results. Generally, choosing a value close to the input data dimension of the deep autoencoder can help achieve better results.

Additionally, i is the index of the data sequence to be filtered corresponding to the starting point of the filtering window, $i_{c}$ is the index of the center point of the window, and $i_{e}$ is the index of the end point of the window.

Step 2: Calculate the average value of the data within the filtering window ${\overset{-}{x}}_{W}$ using the following formula:

(13) $${\overset{-}{x}}_{W}=\frac{1}{N_{W}}\sum_{k=i}^{i_{e}}x_{k}$$

Step 3: Calculate the standard deviation of data within the window $s\left(i_{c}\right)$ using the following formula:

(14) $$s\left(i_{c}\right)=\sqrt{\frac{\sum_{k=i}^{i_{e}}{\left(x_{i}-{\overset{-}{x}}_{W}\right)}^{2}}{N_{W}}}$$

Step 4: Obtain the value of the center point within the filtering window with the following formula:

(15) $$x_{i_{c}\;}=\left\{\begin{array}{c} {\overset{-}{x}}_{W},s\left(i_{c}\right)<T_{W}\\ x_{i_{c}},s\left(i_{c}\right)\geq T_{W} \end{array}\right.$$

Step 5: Output the value of $x_{i_{c}}$ to replace the data value with index $i_{c}$ in the sequence to be filtered, while keeping the data values at other positions unchanged.

Step 6: Move the window index backward by the following formula:

(16) $$i^{\prime }=i+i_{\mathrm{steplen}}$$

where $i^{\prime }$ is the start point index in new window, and $i_{\mathrm{steplen}}$ is the sliding window step length. Replace i with $i^{\prime }$ and continue to perform the mean filtering according to Equations (11)–(15) until all the required data are filtered.

### 2.5. Performance Evaluation

To objectively evaluate the compression performance of the method proposed in this paper, the signal-to-noise ratio (SNR), mean squared error (MSE), compression ratio (CR), and their incremental ratios (ΔSNR, ΔMSE, and ΔCR) are used to evaluate the performance of compression/reconstruction methods. The SNR is calculated as

(17) $$\mathrm{SNR}\left(\mathrm{dB}\right)=10\cdot \log_{10}\left(\frac{\sum_{i=1}^{n}{y_{i}}^{2}}{\sum_{i=1}^{n}{\left(y_{i}-{\hat{y}}_{i}\right)}^{2}}\right)$$

MSE is calculated as

(18) $$\mathrm{MSE}=\frac{1}{n}\sum_{i=1}^{n}{{(\hat{y}}_{i}-y_{i})}^{2}$$

where $y_{i}$ and ${\hat{y}}_{i}$ represent the raw and reconstructed data values, respectively.

The smaller the MSE or the larger the SNR, the smaller the decoding data error, indicating better decoding data quality. When the MSE or SNR is constant, the larger the compression ratio, the better the data compression effect, and the greater the effect on improving the equivalent transmission rate of the MPT system. The compression ratio formula is defined as

(19) $$C_{R}=\frac{S_{O}}{S_{C}}$$

where $S_{O}$ is the amount of raw data, and $S_{C}$ is the amount of compressed data.

ΔSNR is the incremental ratio of SNR and is used to measure the degree of improvement in SNR between two different compression methods. The formula for ΔSNR is as follows:

(20) $${∆\mathrm{SNR}}_{A2,A1}=\frac{{\mathrm{SNR}}_{A2}-{\mathrm{SNR}}_{A1}}{{\mathrm{SNR}}_{A1}}\times 100\%$$

where ${∆\mathrm{SNR}}_{A2,A1}$ represents the percentage of improvement in SNR of method A2 relative to method A1.

The corresponding ΔMSE and ΔCR are the incremental ratios of MSE and CR, respectively, used to measure the degree of performance improvement between different compression methods.

The formulas for ΔMSE and ΔCR are as follows:

(21) $${∆\mathrm{MSE}}_{A2,A1}=\frac{{\mathrm{MSE}}_{A2}-{\mathrm{MSE}}_{A1}}{{\mathrm{MSE}}_{A1}}\times 100\%$$

(22) $${∆CR}_{A2,A1}=\frac{{CR}_{A2}-{CR}_{A1}}{{CR}_{A1}}\times 100\%$$

where ${∆\mathrm{MSE}}_{A2,A1}$ represents the percentage increase or decrease in MSE of method A2 relative to method A1, and ${∆\mathrm{CR}}_{A2,A1}$ represents the percentage increase or decrease in CR of method A2 relative to method A1.

### 2.6. Implementation of the Proposed Method

The implementation process of the proposed method is divided into three stages: training, compression, and decompression.

In the training phase, the deep autoencoder is first trained to achieve efficient compression of the input data. Then, we use the RMSProp optimization algorithm to find the optimal standard deviation threshold required for the mean filtering method. The compression stage utilizes the trained deep autoencoder along with residual data compression methods to compress the measured data. In the decompression stage, the compressed data are decompressed using the deep autoencoder and residual data decompression methods. Subsequently, the reconstructed data are filtered using the optimal standard deviation threshold and mean filtering method to obtain the final decompressed data.

Below are the implementation processes of these three stages.

#### 2.6.1. Training Process

The training process of the proposed method consists of two parts: one is the process of training the deep autoencoder, and the other is the process to find the optimal standard deviation threshold for the mean filter with the RMSProp algorithm.

The process of training the deep autoencoder is as follows (Algorithm 1).
**Algorithm 1:** Training steps for the deep autoencoder$\mathrm{Step}\;1:\;\mathrm{Collect}\;\mathrm{previously}\;\mathrm{measured}\;\mathrm{inclination}\;\mathrm{and}\;\mathrm{azimuth}\;\mathrm{data}\;\mathrm{separately}\;\mathrm{as}\;\mathrm{the}\;\mathrm{training}\;\mathrm{dataset}\;X_{\mathrm{train}}$. $\mathrm{Step}\;2:\;\mathrm{Set}\;\mathrm{the}\;\mathrm{input}\;\mathrm{data}\;\mathrm{dimension}\;D_{\mathrm{in}}$$\;\mathrm{and}\;\mathrm{change}\;\mathrm{the}\;\mathrm{shape}\;\mathrm{of}\;\mathrm{the}\;\mathrm{dataset}\;X_{\mathrm{train}}$$\;\mathrm{to}\;\mathrm{include}\;D_{\mathrm{in}}$ sampling data in each sample. Then, set shuffle to “True” to shuffle the sample order. Step 3: Use MSE as the loss function and the optimization method of Adam to train the deep autoencoder with the structure shown in Table 1. $\mathrm{Step}\;4:\;\mathrm{When}\;\mathrm{the}\;\mathrm{training}\;\mathrm{converges},\;\mathrm{save}\;\mathrm{the}\;\mathrm{corresponding}\;\mathrm{deep}\;\mathrm{autoencoder}\;\mathrm{model}\;\mathrm{parameter}\;P_{\mathrm{model}}$.

The process to find the optimal standard deviation threshold for the mean filter is as follows (Algorithm 2).
**Algorithm 2:** Steps to find the optimal standard deviation thresholdStep 1: Load the deep autoencoder shown in Table 1$\;\mathrm{and}\;\mathrm{model}\;\mathrm{parameter}\;P_{\mathrm{model}}$$,\;\mathrm{use}\;\mathrm{the}\;\mathrm{deep}\;\mathrm{autoencoder}\;\mathrm{to}\;\mathrm{compress}\;\mathrm{and}\;\mathrm{decompress}\;\mathrm{the}\;\mathrm{dataset}\;X_{\mathrm{train}}$$,\;\mathrm{and}\;\mathrm{obtain}\;\mathrm{compressed}\;\mathrm{data}\;X_{\mathrm{train}\text{\_}\mathrm{com}}$$\;\mathrm{and}\;\mathrm{decompressed}\;\mathrm{data}\;X_{\mathrm{train}\text{\_}\mathrm{dec}}$. $\mathrm{Step}\;2:\;\mathrm{Subtract}\;\mathrm{the}\;\mathrm{autoencoder}\;\mathrm{decompressed}\;\mathrm{data}\;X_{\mathrm{train}\text{\_}\mathrm{dec}}$$\;\mathrm{from}\;\mathrm{the}\;\mathrm{original}\;\mathrm{dataset}\;X_{\mathrm{train}}$$\;\mathrm{to}\;\mathrm{obtain}\;\mathrm{the}\;\mathrm{residual}\;\mathrm{data}\;X_{\mathrm{train}\text{\_}\mathrm{res}}$. Step 3: Set the quantization bits for quantization coding to $B_{q}$, perform quantization coding on the residual data $X_{\mathrm{train}\text{\_}\mathrm{res}}$ according to Formulas (6) and (7), and then perform Huffman coding on the quantized encoded data according to the method in ref. [13] to obtain the compressed data of the residual data $X_{\mathrm{train}\text{\_}\mathrm{re}\mathrm{scom}}$. Step 4: Decode the compressed data of the residual data $X_{\mathrm{train}\text{\_}\mathrm{rescom}}$ with Huffman decoding and quantization decoding according to the inverse process of the method in step 3 to obtain the reconstructed data of the residual data $X_{\mathrm{train}\text{\_}\mathrm{res}}^{\prime }$. Step 5: Add the reconstructed residual data $X_{\mathrm{train}\text{\_}\mathrm{res}}^{\prime }$ and decompressed data $X_{\mathrm{train}\text{\_}\mathrm{dec}}$ to obtain the reconstructed test dataset $X_{\mathrm{train}}^{\prime }$. Step 6: Use the RMSProp algorithm (in Section 2.4.1 of this paper) to find the optimal standard deviation threshold $T_{W}$. The detailed steps are as follows:
(1)Set values for hyperparameters β, lr, and ε$.\;\mathrm{Set}\;\mathrm{the}\;\mathrm{standard}\;\mathrm{deviation}\;\mathrm{threshold}\;T\;\mathrm{to}\;\mathrm{its}\;\mathrm{initial}\;\mathrm{value}\;T_{0}$$.\;\mathrm{Set}\;\mathrm{the}\;\mathrm{mean}\;\mathrm{filtering}\;\mathrm{window}\;\mathrm{size}\;\mathrm{to}\;N_{W}$ and the step length to StepLen.(2)$\mathrm{Starting}\;\mathrm{from}\;\mathrm{the}\;\mathrm{first}\;\mathrm{data},\;\mathrm{take}\;N_{W}$$\;\mathrm{data}\;\mathrm{points}\;\mathrm{in}\;\mathrm{order}\;\mathrm{from}\;\mathrm{the}\;\mathrm{reconstructed}\;\mathrm{data}\;X_{\mathrm{train}}^{\prime }$ and fill them in the filtering window.(3)Use the standard deviation threshold T, filter the center point data of the filtering window according to formulas (11) to (15), and output the center point data of the window.(4)According to Equation (16), move the filtering window backward according to the step length StepLen,$\;\mathrm{and}\;\mathrm{then}\;\mathrm{take}\;N_{W}$ data points in order to fill the filtering window.
(5)$\mathrm{Repeat}\;\mathrm{steps}\;(3)\;\mathrm{to}\;(4)\;\mathrm{until}\;\mathrm{the}\;\mathrm{remaining}\;\mathrm{number}\;\mathrm{of}\;\mathrm{data}\;\mathrm{is}\;\mathrm{less}\;\mathrm{than}\;N_{W}$$,\;\mathrm{and}\;\mathrm{obtain}\;\mathrm{the}\;\mathrm{filtered}\;\mathrm{data}\;X_{\mathrm{filter}}^{\prime }$.
(6)$\mathrm{Calculate}\;\mathrm{the}\;\mathrm{MSE}\;\mathrm{of}\;\mathrm{the}\;\mathrm{original}\;\mathrm{data}\;X_{\mathrm{train}}$$\;\mathrm{and}\;\mathrm{the}\;\mathrm{filtered}\;\mathrm{data}\;X_{\mathrm{filter}}^{\prime }$ as the loss function, and use Equation (10) to calculate the gradient value of this on the standard deviation threshold T.
(7)Use the gradient value from step (6) to update the standard deviation threshold according to Equations (8) and (9).(8)$\mathrm{Repeat}\;\mathrm{steps}\;(2)\;\mathrm{to}\;(7),\;\mathrm{and}\;\mathrm{perform}\;\mathrm{multiple}\;\mathrm{iterations}\;\mathrm{to}\;\mathrm{update}\;\mathrm{until}\;\mathrm{the}\;\mathrm{loss}\;\mathrm{function}\;\mathrm{converges}\;\mathrm{to}\;a\;\mathrm{certain}\;\mathrm{value}\;\mathrm{and}\;\mathrm{the}\;\mathrm{optimal}\;\mathrm{standard}\;\mathrm{deviation}\;\mathrm{threshold}\;T_{W}$ is obtained.

Step 7: Output the optimal standard deviation threshold $T_{W}$.

#### 2.6.2. Data Compression Process

The data compression process is as follows (Algorithm 3).
**Algorithm 3:** Steps for compression encodingStep 1: Load the deep autoencoder model with the trained model parameters $P_{\mathrm{model}}$. Step 2: Collect $D_{\mathrm{in}}$ data points as a set of data X, and input X into the encoder of the deep autoencoder to obtain the compressed data, denoted as $X_{\mathrm{com}}$. Step 3: Input $X_{\mathrm{com}}$ into the decoder of the deep autoencoder to obtain the decompressed data $X_{\mathrm{dec}}$, and subtract $X_{\mathrm{dec}}$ from X to obtain the residual data $X_{\mathrm{res}}$. Step 4: Encode the residual data $X_{\mathrm{res}}$ with quantization coding and Huffman coding to obtain the compressed data of the residual data $X_{\mathrm{rcom}}.$ Step 5: Output the compressed data of X ($X_{\mathrm{com}}$) and compressed data of residual data ($X_{\mathrm{rcom}}$). Step 6: Repeat steps 2 to 5 until all the collected data are completed.

#### 2.6.3. Data Decompression Process

The data decompression process is as follows (Algorithm 4).
**Algorithm 4:** Steps for decompression$\mathrm{Step}\;1:\;\mathrm{Load}\;\mathrm{the}\;\mathrm{deep}\;\mathrm{autoencoder}\;\mathrm{model}\;\mathrm{with}\;\mathrm{the}\;\mathrm{trained}\;\mathrm{model}\;\mathrm{parameter}\;P_{\mathrm{model}}$$,\;\mathrm{set}\;\mathrm{the}\;\mathrm{mean}\;\mathrm{filtering}\;\mathrm{window}\;\mathrm{size}\;\mathrm{to}\;N_{W}$ and the step length to StepLen$,\;\mathrm{and}\;\mathrm{use}\;\mathrm{the}\;\mathrm{optimal}\;\mathrm{threshold}\;T_{W}$ obtained from Algorithm 2 as the standard deviation threshold required for the mean filtering method. $\mathrm{Step}\;2:\;\mathrm{Obtain}\;\mathrm{the}\;\mathrm{compressed}\;\mathrm{data}\;(X_{\mathrm{com}}$$)\;\mathrm{and}\;\mathrm{the}\;\mathrm{compressed}\;\mathrm{data}\;\mathrm{of}\;\mathrm{residual}\;\mathrm{data}\;(X_{\mathrm{rcom}}$). $\mathrm{Step}\;3:\;\mathrm{Input}\;\mathrm{the}\;\mathrm{compressed}\;\mathrm{data}\;(X_{\mathrm{com}}$$)\;\mathrm{into}\;\mathrm{the}\;\mathrm{decoder}\;\mathrm{of}\;\mathrm{the}\;\mathrm{deep}\;\mathrm{autoencoder}\;\mathrm{to}\;\mathrm{obtain}\;\mathrm{the}\;\mathrm{decompressed}\;\mathrm{data}\;(X_{\mathrm{dec}}$). $\mathrm{Step}\;4:\;\mathrm{Decode}\;\mathrm{the}\;\mathrm{compressed}\;\mathrm{data}\;(X_{\mathrm{rcom}}$$)\;\mathrm{with}\;\mathrm{Huffman}\;\mathrm{decoding}\;\mathrm{and}\;\mathrm{quantization}\;\mathrm{decoding}\;\mathrm{to}\;\mathrm{obtain}\;\mathrm{the}\;\mathrm{reconstructed}\;\mathrm{residual}\;\mathrm{data}\;(X_{\mathrm{res}}^{\prime }$). $\mathrm{Step}\;5:\;\mathrm{Add}\;\mathrm{the}\;\mathrm{decompressed}\;\mathrm{data}\;(X_{\mathrm{dec}}$$)\;\mathrm{and}\;\mathrm{the}\;\mathrm{reconstructed}\;\mathrm{residual}\;\mathrm{data}\;(X_{\mathrm{res}}^{\prime }$$)\;\mathrm{to}\;\mathrm{obtain}\;\mathrm{the}\;\mathrm{reconstructed}\;\mathrm{collected}\;\mathrm{data}\;(X^{\prime }$). $\mathrm{Step}\;6:\;\mathrm{Take}\;N_{W}$$\;\mathrm{data}\;\mathrm{points}\;\mathrm{of}\;X^{\prime }$ in order and fill them in the filtering window. $\mathrm{Step}\;7:\;\mathrm{Use}\;\mathrm{the}\;\mathrm{optimal}\;\mathrm{standard}\;\mathrm{deviation}\;\mathrm{threshold}\;T_{W}$ and step length StepLen$,\;\mathrm{filter}\;\mathrm{the}\;\mathrm{data}\;\mathrm{in}\;X^{\prime }$ according to Equations (11)–(16), and output the filtered data.Step 8: Repeat steps 2 to 7 until all the compressed data have been decoded.

## 3. Experiments

### 3.1. Experimental Data

**Figure 5:**
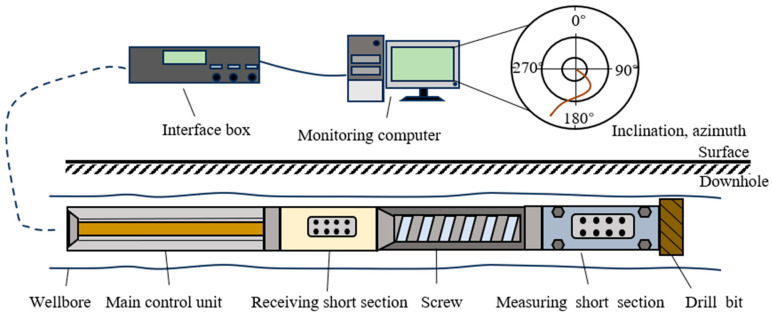
Schematic diagram of LWD system device operation.

To evaluate the proposed method, a dataset comprising inclination and azimuth data obtained from an LWD system was collected for experimentation. The schematic diagram of the LWD system is depicted in Figure 5. The measurement short section in the downhole component includes an inclination and azimuth measurement module, which captures information on the well’s inclination and azimuth near the drill bit. This information is transmitted to the receiving short section via an antenna, encoded by the main control unit, and then sent to the ground. Finally, it is decoded by the interface box and monitoring computer in the ground section to provide inclination and azimuth data for monitoring the stable state of the wellbore by ground workers.

The monitoring computer records the decoded inclination and azimuth data while monitoring. We obtained the inclination and azimuth data of multiple wells measured at different time periods recorded by this LWD system, denoted as $X_{\mathrm{train}\text{\_}\mathrm{test}}$. The $X_{\mathrm{train}\text{\_}\mathrm{test}}$ contains 72,328 points each for the inclination and azimuth data, with 80% (57,864 sampling data) of the data taken as the training dataset, denoted as the inclination training dataset $X_{\mathrm{inc}\text{\_}\mathrm{train}}$ and azimuth training dataset $X_{\mathrm{azi}\text{\_}\mathrm{train}}$. The remaining 20% (14,464 sampling data) will be used as the testing dataset, denoted as the inclination testing dataset $X_{\mathrm{inc}\text{\_}\mathrm{test}}$ and azimuth testing dataset $X_{\mathrm{azi}\text{\_}\mathrm{test}}$. Among them, $X_{\mathrm{inc}\text{\_}\mathrm{train}}$ and $X_{\mathrm{azi}\text{\_}\mathrm{train}}$ are used for Algorithm 1 to train the deep autoencoder, and for Algorithm 2 to obtain the optimal standard deviation threshold of the mean filter. In addition, $X_{\mathrm{inc}\text{\_}\mathrm{test}}$ and $X_{\mathrm{azi}\text{\_}\mathrm{test}}$ are used to test and compare the compression performance of various compression methods.

### 3.2. Experimental Setup

To verify the effectiveness of the proposed method in this paper, we first use the datasets of $X_{\mathrm{inc}\text{\_}\mathrm{train}}$ and $X_{\mathrm{azi}\text{\_}\mathrm{train}}$ to train the deep autoencoder shown in Table 1 with Algorithm 1. The dimension of the input data ($D_{\mathrm{in}}$) of the deep autoencoder is set to 8. After the training, the deep autoencoder models are obtained for inclination data and azimuth data. Algorithm 2 and training datasets $X_{\mathrm{inc}\text{\_}\mathrm{train}}$ and $X_{\mathrm{azi}\text{\_}\mathrm{train}}$ are used to find the optimal standard deviation thresholds for inclination data and azimuth data, respectively. The window size $N_{W}$ of the mean filter in Algorithm 2 is set to 5, and the initial value of the standard deviation threshold is flexibly selected based on the convergence situation. The quantization bits are set to 4, 5, 6, 7, and 8, respectively, to obtain the optimal filtering threshold $T_{W}$ for different compression multiples and errors.

## 4. Analysis and Discussion of the Experimental Results

To verify the effectiveness of the method proposed in this paper, the following compression methods were used for comparative experiments:

DeepAE+QC+HC+F (the proposed method): The wellbore stability monitoring data compression method based on deep autoencoder, consisting of the deep autoencoder (deepAE), quantization coding (QC), Huffman coding (HC), and the mean filtering method based on optimal standard deviation threshold (F).

DPCM: The DPCM method in ref. [23]. This method uses the simplest first-order predictor to compress LWD data.

DPCM-I: The DPCM method in ref. [24]. This method requires the use of previously measured LWD data, using the minimum mean square error as a criterion to determine the optimal predictor parameters, and then using these parameters to compress the current data that need to be compressed. Its performance is stronger than that of the DPCM method.

deepAE: The deep autoencoder method. Its structure adopts the structure shown in Table 1, and it is trained using Algorithm 1 in Section 2.6.1 and datasets $X_{\mathrm{inc}\text{\_}\mathrm{train}}$ and $X_{\mathrm{azi}\text{\_}\mathrm{train}}$.

deepAE+QC+HC: The deep autoencoder (deepAE), combining the quantization encoding (QC) and Huffman coding (HC) methods. Firstly, use the deep autoencoder to compress the data, then perform quantization encoding and Huffman coding on the obtained residual data. Finally, compensate for the reconstructed data in the decoder section of the deep autoencoder.

All deepAE methods mentioned above are the same, have the same structure, and use the same training dataset for training, making it easier to verify the functionality of different components of the proposed method.

### 4.1. Data Feature Extraction Results

**Figure 6:**
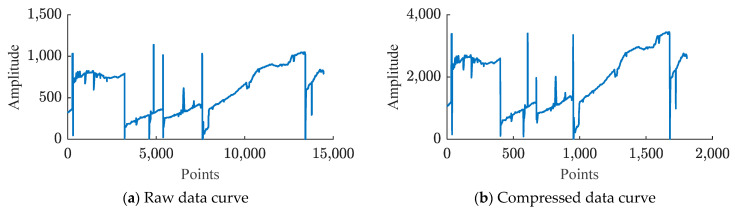

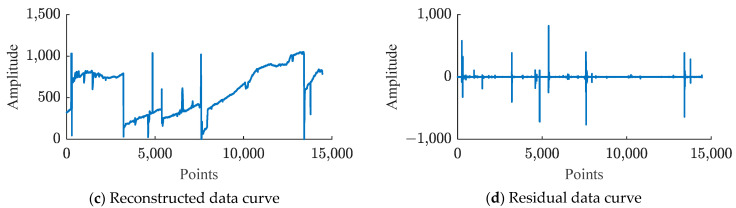
Compression results of deep autoencoder on dataset $X_{\mathrm{inc}\text{\_}\mathrm{test}}$: (**a**) represents the original data of $X_{\mathrm{inc}\text{\_}\mathrm{test}}$ and $X_{\mathrm{azi}\text{\_}\mathrm{test}}$, (**b**) represents the features extracted by the deep autoencoder, (**c**) represents the reconstructed data of the deep autoencoder, and (**d**) represents the residual between the raw data and the reconstructed data.

**Figure 7:**
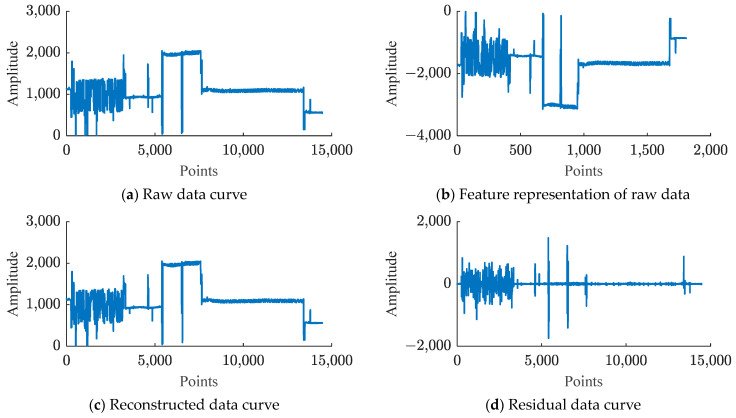
Compression results of deep autoencoder on dataset $X_{\mathrm{azi}\text{\_}\mathrm{test}}$: (**a**) represents the original data of $X_{\mathrm{inc}\text{\_}\mathrm{test}}$ and $X_{\mathrm{azi}\text{\_}\mathrm{test}}$, (**b**) represents the features extracted by the deep autoencoder, (**c**) represents the reconstructed data of the deep autoencoder, and (**d**) represents the residual between the raw data and the reconstructed data.

The compression results of $X_{\mathrm{inc}\text{\_}\mathrm{test}}$ and $X_{\mathrm{azi}\text{\_}\mathrm{test}}$ data using a trained deep autoencoder are shown in Figure 6 and Figure 7.

In Figure 6 and Figure 7, due to the input data dimension $D_{\mathrm{in}}$ of the deep autoencoder being 8, according to the structure of the deep autoencoder in Table 1, the bottleneck layer has a dimension of 1. Hence the extracted features have a dimension of 1 after compression. Therefore, (b) in Figure 6 and Figure 7 show all the features extracted by the deep autoencoder. Comparing (a) and (b) in Figure 6 and Figure 7 reveals that although the number of points in the proposed feature data is smaller than that of the raw data, its shape is very similar or symmetrical to the raw data, indicating that the deep autoencoder effectively extracts the main features of the raw data. The (d) figures in Figure 6 and Figure 7 illustrate that after feature extraction by a deep autoencoder, there are still small differences between the reconstructed data and the original data. Therefore, using quantization coding and Huffman coding to compress the residuals will help reduce the error between the reconstructed data and the source data.

### 4.2. Comparison of Compression Results

We used the proposed method, deepAE, DPCM, and DPCM-I to compress $X_{\mathrm{inc}\text{\_}\mathrm{test}}$ and $X_{\mathrm{azi}\text{\_}\mathrm{test}}$.

Due to the input dimension of the proposed method and deepAE method, the shape of the $X_{\mathrm{inc}\text{\_}\mathrm{test}}$ and $X_{\mathrm{azi}\text{\_}\mathrm{test}}$ data was reset to (18,088). In addition to the deepAE method, the quantization data bits of the proposed method, DPCM method, and DPCM-I method were set to 4, 5, 6, 7, and 8 to verify the error reduction performance under different quantization data bits. The compression results are shown in Table 2.

$X_{\mathrm{inc}\text{\_}\mathrm{test}}$$X_{\mathrm{azi}\text{\_}\mathrm{test}}$$X_{\mathrm{inc}\text{\_}\mathrm{test}}$$X_{\mathrm{azi}\text{\_}\mathrm{test}}$$X_{\mathrm{inc}\text{\_}\mathrm{test}}$$X_{\mathrm{azi}\text{\_}\mathrm{test}}$$X_{\mathrm{inc}\text{\_}\mathrm{test}}$$X_{\mathrm{azi}\text{\_}\mathrm{test}}$ According to Table 2, to verify the performance improvement of the proposed method, we used Formulas (20)~(22) to calculate the compression ratio increment ratio (ΔCR), signal-to-noise ratio increment ratio (ΔSNR), and mean square error increment ratio (ΔMSE) of the proposed method relative to deepAE, DPCM, and DPCM-I. The results are shown in Table 3.

According to Table 2 and Table 3, it can be seen that compared to the DPCM and DPCM-I methods, the deep autoencoder (deepAE) shows a significant improvement in compression ratio (CR), reaching 7.33, but its error is also significant, with a signal-to-noise ratio (SNR) of only 25.25 dB and a mean square error (MSE) of 4122.73. On the basis of deepAE, our proposed method was enhanced. Although the compression ratio was reduced by 44.69%, the error was significantly reduced, the signal-to-noise ratio was improved by 79.73%, and the mean square error was reduced by 97.65%. The proposed method maintains high compression performance while significantly reducing errors, thus achieving a better balance.

In addition, it can be seen that under the same number of quantization bits, the compression ratio and signal-to-noise ratio of the proposed method are overwhelmingly higher than those of DPCM method and DPCM-I method, while MSE is overall lower than those of DPCM and DPCM-I. Among them, under the quantization bits of 4~8 bits, the compression ratio of the proposed method for the inclination angle data $X_{\mathrm{inc}\text{\_}\mathrm{test}}$ and azimuth data $X_{\mathrm{azi}\text{\_}\mathrm{test}}$ is 3.22~4.38, with an average compression ratio of 4.05. Compared to DPCM and DPCM-I, the proposed method improves the compression ratio by 53.80~203.78%, with an average improvement of 118.54%. Meanwhile, the signal-to-noise ratio of the proposed method reached 35.34~53.22 dB, with an average of 45.09 dB; compared to DPCM-I, it is improved by 6.46~81.39%, with an average improvement of 32.40%. Compared to DPCM, the signal-to-noise ratio is improved by 7.78~84.09%, with an average improvement of 35.84%. The mean square error of the proposed method reached 1.98~428.61, with an average of 76.88; compared to DPCM-I, it decreased by 51.76~98.00%, with an average decrease of 82.46%. Compared with DPCM, it decreased by 57.98~98.21%, with an average decrease of 86.31%. These results indicate that the compression ratio of the proposed method is significantly improved compared to the DPCM and DPCM-I, and the error of the reconstructed data is significantly reduced.

**Figure 8:**
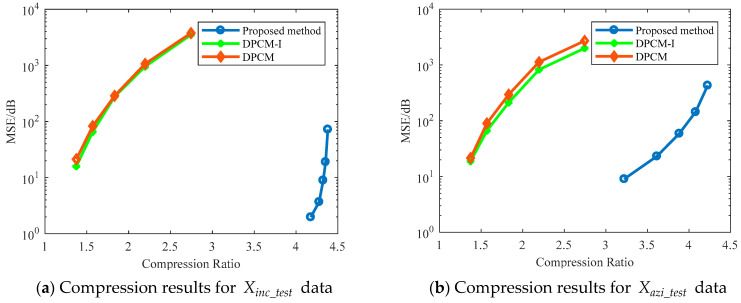
Comparison of compression performance between the proposed method, DPCM-I, and DPCM: (**a**) represents the compression results for $X_{\mathrm{inc}\text{\_}\mathrm{test}}$ data, (**b**) represents the compression results for $X_{\mathrm{azi}\text{\_}\mathrm{test}}$ data.

Figure 8 compares the compression performance between the proposed method, DPCM-I, and DPCM. It is evident that the proposed method generally achieves a higher compression ratio than DPCM-I and DPCM, while also exhibiting a lower overall mean square error (MSE). Even in cases where the MSE is similar, the proposed method consistently achieves a higher compression ratio, indicating superior compression performance.

**Figure 9:**
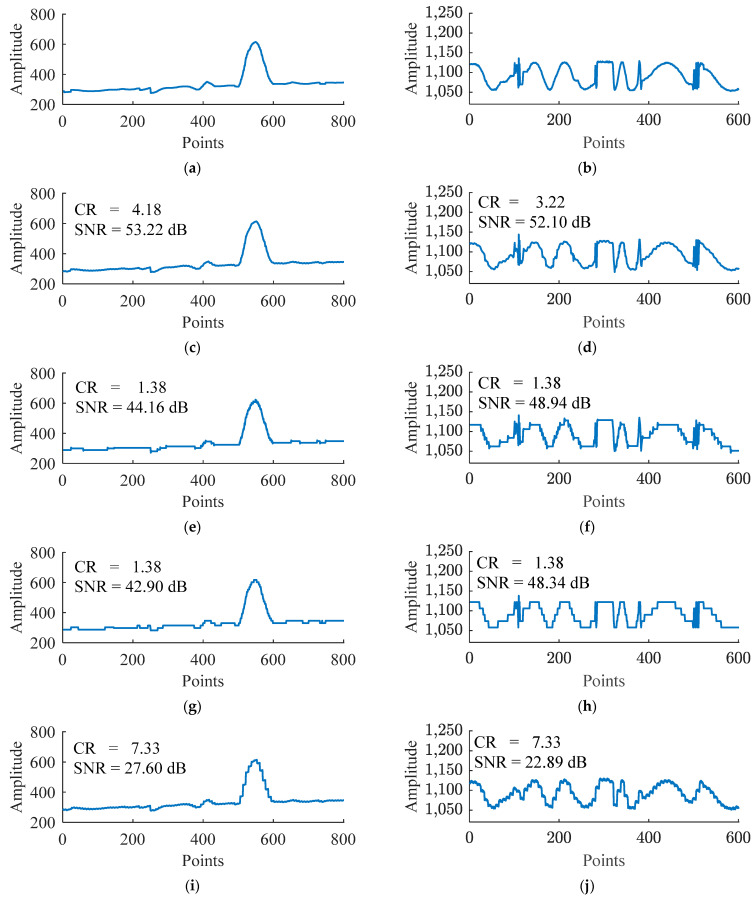
Comparison of the reconstructed data of the proposed method, deepAE, DPCM-I, and DPCM and the raw data: (**a**,**b**) represent a portion of the original data curves in $X_{\mathrm{inc}\text{\_}\mathrm{test}}$ and $X_{\mathrm{azi}\text{\_}\mathrm{test}}$, respectively. (**c**,**e**,**g**,**i**) represent the reconstructed data corresponding to (**a**) after compressing and decompressing $X_{\mathrm{inc}\text{\_}\mathrm{test}}$ data using the proposed method, DPCM-I, DPCM and deepAE, respectively; (**d**,**f**,**h**,**j**) represent the reconstructed data corresponding to (**b**) after compressing and decompressing $X_{\mathrm{azi}\text{\_}\mathrm{test}}$ data using the proposed method, DPCM-I, DPCM, and deepAE, respectively.

To intuitively demonstrate the compression performance of the proposed method, deepAE, DPCM-I, and DPCM, the reconstructed data curves of the inclination data ($X_{\mathrm{inc}\text{\_}\mathrm{test}}$) and azimuth data ($X_{\mathrm{azi}\text{\_}\mathrm{test}}$) using these methods under 8-bit quantization are plotted and compared with the original data curves. The results are shown in Figure 9.

In Figure 9, by observing and comparing Figure 9a,c,e,g,i, it can be seen that the reconstructed data curve of the proposed method is closest to the original data curve, followed by DPCM-I and then DPCM, while the reconstructed data curve of deepAE has the largest difference between the reconstructed data curve and the original data curve, which is consistent with the compression ratio and signal-to-noise ratio reflected in Figure 9c,e,g,i. Similarly, by observing and comparing Figure 9b,d,f,h,j, the same conclusion can be drawn. The above results indicate that compared with the DPCM methods, the proposed method in this paper has a higher compression ratio for inclination and azimuth data, and at the same time, it has a smaller error in reconstructing the data curve, which is more in line with the original data curve.

### 4.3. Ablation Study

To quantitatively ascertain the contributory impact of individual components within the proposed method, a meticulously structured series of ablation study analyses were undertaken. The purpose of these analyses is to demonstrate the effective role of newly integrated components in terms of compression performance or reducing errors. Among them, for methods containing quantization coding, the quantization data bits are set to 4, 5, 6, 7, and 8 to fully verify the compression performance of these methods under different quantization data bits. Table 4 presents the results of the ablation experiment.

In the ablation study, the benchmark model selected was the deepAE method; as shown in Table 4, the addition of QC+HC and F continuously reduced the error of the reconstructed data of the benchmark model, while the compression ratio was still at a relatively high level.

By introducing QC+HC, the mean square error was reduced from 4122.73 to 91.13, a decrease of 97.79%. Although the compression ratio decreased from 7.33 to 4.06 (a decrease of 44.61%), as shown in Table 3, the current compression ratio was 118.54% higher than the DPCM-I method and still at a relatively high level.

By introducing F, the mean square error was further reduced to 76.87, which is 98.14% lower than the benchmark model. The proposed mean filter based on optimal standard deviation threshold effectively further reduced errors.

The ablation experiment conducted shows that the proposed combination effectively reduces errors while maintaining a high level of compression ratio. Therefore, the proposed method achieves a better balance between compression performance and error compared to the benchmark model.

**Figure 10:**
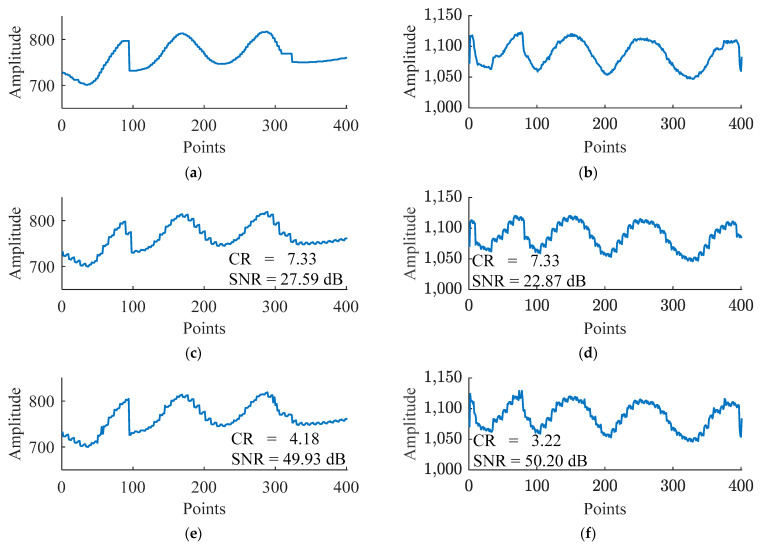

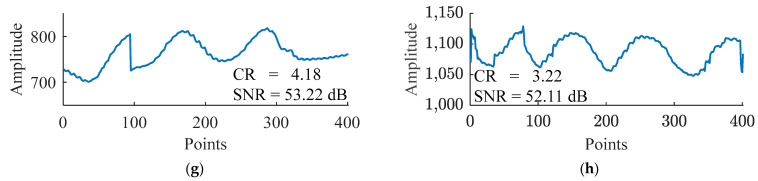
Comparison of differences between the reconstructed data curves of deepAE, deepAE+QC+HC, and the proposed method and the original data curves: (**a**,**b**) represent a portion of the original data curves in $X_{\mathrm{inc}\text{\_}\mathrm{test}}$ and $X_{\mathrm{azi}\text{\_}\mathrm{test}}$, respectively; (**c**,**e**,**g**) represent the reconstructed data corresponding to (**a**) after compressing and decompressing $X_{\mathrm{inc}\text{\_}\mathrm{test}}$ data using deepAE, deepAE+QC+HC, and the proposed method, respectively; (**d**,**f**,**h**) represent the reconstructed data corresponding to (**b**) after compressing and decompressing $X_{\mathrm{azi}\text{\_}\mathrm{test}}$ data using deepAE, deepAE+QC+HC, and the proposed method, respectively.

To more intuitively demonstrate the progressive effect of each component of the proposed method on reducing the error between reconstructed data and original data, the reconstructed data curves of the deepAE method, the deepAE+QC+HC method, and the proposed method (deepAE+QC+HC+F) on $X_{\mathrm{inc}\text{\_}\mathrm{test}}$ and $X_{\mathrm{azi}\text{\_}\mathrm{test}}$ data were plotted and compared with the original data curves. The results are shown in Figure 10.

In Figure 10, by comparing Figure 10a,c,e,g, it can be seen that the reconstructed data curve of the deepAE method shows the greatest difference from the original data curve, followed by the deepAE+QC+HC method. The reconstructed data curve of our proposed method is closest to the original data curve, which is consistent with the signal-to-noise ratio reflected in Figure 10c,e,g. By comparing Figure 10b,d,f,h, the same conclusion can be drawn. The above results indicate that as the QC+HC component and F component are sequentially added to the benchmark model deepAE, the error of reconstructed data becomes smaller and closer to the original data curve. This result further demonstrates that our proposed method effectively reduces the error of the reconstructed data and the original data and maintains a high level of compression ratio, achieving a better balance.

The above results indicate that our proposed method effectively improves the compression ratio and reduces reconstruction data errors, which is more conducive to improving the performance of wellbore stability monitoring and achieving the goal of improving production safety.

## 5. Conclusions

This paper introduced a novel compression method utilizing a deep autoencoder to significantly enhance the compression ratio of inclination and azimuth data during drilling operations. Additionally, residual data were encoded using quantization coding and Huffman coding, effectively minimizing the error between reconstructed data and source data. Moreover, a mean filtering technique based on the optimal standard deviation threshold was employed to further mitigate the error between the reconstructed data and the source data. Simulation testing on inclination and azimuth data demonstrates that the average compression ratio of the proposed method is 4.05; compared to the DPCM methods, it is improved by 118.54%. Meanwhile, the average mean square error of the proposed method is 76.88, which is decreased by 82.46% when compared to the DPCM method. The results of the ablation experiment also indicate that our method achieves a better balance between compression performance and error compared to deep autoencoders.

This work pioneers the use of a deep autoencoder-based methods for compressing wellbore trajectory data and integrates classical compression and filtering methods to effectively process residual and reconstructed data, thereby enhancing compression performance and reducing errors between reconstructed data and source data. The compression method proposed in this paper for wellbore stability monitoring data has real-time performance, a higher compression ratio, and a smaller reconstruction data error, solving the problems of low real-time performance, low compression ratio, and large error in the existing methods. This not only provides valuable insights for the advancement of data compression technology related to LWD data but also holds significant implications for enhancing the safety monitoring performance of wellbores in logging for drilling engineering.

Considering the deep autoencoder’s excellent capability in feature extraction and the fact that the proposed method in this paper integrates neural networks with classical data compression techniques, the proposed method holds reference value for the study of other types of LWD data compression methods. Additionally, it also provides a certain degree of reference significance for the research of data compression methods in other fields.

Although the proposed method effectively improves the performance of wellbore stability monitoring, the method only discusses a simple deep autoencoder structure. In other fields, such as image data compression, many excellent neural network structures have been used for data compression. Therefore, future research can focus on the exploration and innovation of neural network structures to further improve the compression performance and reduce the error of the reconstructed data and the original data.

## Figures and Tables

**Table 1:** Structure of the deep autoencoder used in the proposed method.

Composition	Layer Name	Layer Type	Activation Function	Dimension
Encoder	input layer	-	-	*n*
	hidden layer 1	Dense	ϕ=leaky_relu	*n/2*
	hidden layer 2	Dense	ϕ=leaky_relu	*n/4*
	bottleneck	Dense	ϕ=leaky_relu	$\hat{n}$ * = n/8*
Decoder	hidden layer 3	Dense	ϕ=leaky_relu	*n/4*
	hidden layer 4	Dense	ϕ=leaky_relu	*n/2*
	hidden layer 5	Dense	ϕ=leaky_relu	*n*
	output layer	-	-	*n*

**Table 2:** Comparison results of the proposed method, deepAE, DPCM, and DPCM-I.

Method	Data Set	Quantization Data Bits	Tw	CR	SNR/dB	MSE
DPCM	$X_{\mathrm{inc}\text{\_}\mathrm{test}}$	4	-	2.75	20.42	3767.45
		5	-	2.20	25.90	1067.62
		6	-	1.83	31.60	286.80
		7	-	1.57	36.99	83.06
		8	-	1.38	42.90	21.29
	$X_{\mathrm{azi}\text{\_}\mathrm{test}}$	4	-	2.75	27.34	2704.75
		5	-	2.20	31.15	1124.74
		6	-	1.83	36.98	293.73
		7	-	1.57	42.13	89.66
		8	-	1.38	48.34	21.47
	Average			1.94	34.37	946.06
DPCM-I	$X_{\mathrm{inc}\text{\_}\mathrm{test}}$	4	-	2.75	20.72	3512.95
		5	-	2.20	26.39	952.75
		6	-	1.83	31.77	276.33
		7	-	1.57	38.03	65.28
		8	-	1.38	44.16	15.92
	$X_{\mathrm{azi}\text{\_}\mathrm{test}}$	4	-	2.75	28.68	1986.52
		5	-	2.20	32.50	824.54
		6	-	1.83	38.42	210.84
		7	-	1.57	43.46	66.09
		8	-	1.38	48.94	18.70
	Average			1.94	35.31	792.99
deepAE	$X_{\mathrm{inc}\text{\_}\mathrm{test}}$	-	-	7.33	27.60	721.63
	$X_{\mathrm{azi}\text{\_}\mathrm{test}}$	-	-	7.33	22.89	7523.82
	Average			7.33	25.25	4122.73
The proposed method	$X_{\mathrm{inc}\text{\_}\mathrm{test}}$	4	53.25	4.38	37.59	72.26
		5	45.51	4.35	43.38	19.06
		6	33.61	4.33	46.66	8.96
		7	21.81	4.28	50.50	3.70
		8	11.01	4.18	53.22	1.98
	$X_{\mathrm{azi}\text{\_}\mathrm{test}}$	4	55.23	4.23	35.34	428.61
		5	50.05	4.08	40.10	143.27
		6	24.50	3.88	43.96	58.82
		7	11.79	3.62	48.03	23.05
		8	6.18	3.22	52.10	9.02
	Average			4.05	45.09	76.87

**Table 3:** Performance improvement of the proposed method relative to DPCM, DPCM-I, and deepAE.

Method Used for Comparison	Data Set	Quantization Data Bits	ΔCR	ΔSNR	ΔMSE
DPCM	$X_{\mathrm{inc}\text{\_}\mathrm{test}}$	4	59.48%	84.09%	−98.08%
		5	98.00%	67.52%	−98.21%
		6	136.08%	47.64%	−96.88%
		7	172.25%	36.54%	−95.55%
		8	203.78%	24.06%	−90.70%
	$X_{\mathrm{azi}\text{\_}\mathrm{test}}$	4	53.80%	29.28%	−84.15%
		5	85.54%	28.74%	−87.26%
		6	112.01%	18.88%	−79.98%
		7	130.11%	14.00%	−74.29%
		8	134.33%	7.78%	−57.98%
	Average		118.54%	35.85%	−86.31%
DPCM-I	$X_{\mathrm{inc}\text{\_}\mathrm{test}}$	4	59.48%	81.39%	−97.94%
		5	98.00%	64.38%	−98.00%
		6	136.08%	46.89%	−96.76%
		7	172.25%	32.78%	−94.33%
		8	203.78%	20.52%	−87.56%
	$X_{\mathrm{azi}\text{\_}\mathrm{test}}$	4	53.80%	23.23%	−78.42%
		5	85.54%	23.40%	−82.62%
		6	112.01%	14.43%	−72.10%
		7	130.11%	10.53%	−65.12%
		8	134.33%	6.46%	−51.76%
	Average		118.54%	32.40%	−82.46%
deepAE	$X_{\mathrm{inc}\text{\_}\mathrm{test}}$	4	−40.23%	36.20%	−89.99%
		5	−40.60%	57.17%	−97.36%
		6	−41.00%	69.06%	−98.76%
		7	−41.65%	82.97%	−99.49%
		8	−43.02%	92.83%	−99.73%
	$X_{\mathrm{azi}\text{\_}\mathrm{test}}$	4	−42.36%	54.39%	−94.30%
		5	−44.34%	75.19%	−98.10%
		6	−47.01%	92.05%	−99.22%
		7	−50.68%	109.83%	−99.69%
		8	−56.04%	127.61%	−99.88%
	Average		−44.69%	79.73%	−97.65%

**Table 4:** Ablation experimental results. √ represents that the module is used. ✕ represents that the module is not used.

deepAE	QC+HC	F	Data Set	Quantization Data Bits	Tw	CR	SNR/dB	MSE
√	✕	✕	$X_{\mathrm{inc}\text{\_}\mathrm{test}}$	-	-	7.33	27.60	721.63
			$X_{\mathrm{azi}\text{\_}\mathrm{test}}$	-	-	7.33	22.89	7523.82
			Mean			7.33	25.25	4122.73
√	√	✕	$X_{\mathrm{inc}\text{\_}\mathrm{test}}$	4	-	4.38	36.98	83.25
				5	-	4.35	42.17	25.15
				6	-	4.33	44.90	13.42
				7	-	4.28	48.08	6.45
				8	-	4.18	49.93	4.22
			$X_{\mathrm{azi}\text{\_}\mathrm{test}}$	4	-	4.23	34.86	478.87
				5	-	4.08	39.22	175.22
				6	-	3.88	42.75	77.85
				7	-	3.62	46.49	32.86
				8	-	3.22	50.19	14.01
			Mean			4.06	43.56	91.13
√	√	√	$X_{\mathrm{inc}\text{\_}\mathrm{test}}$	4	53.25	4.38	37.59	72.26
				5	45.51	4.35	43.38	19.06
				6	33.61	4.33	46.66	8.96
				7	21.81	4.28	50.50	3.70
				8	11.01	4.18	53.22	1.98
			$X_{\mathrm{azi}\text{\_}\mathrm{test}}$	4	55.23	4.23	35.34	428.61
				5	50.05	4.08	40.10	143.27
				6	24.50	3.88	43.96	58.82
				7	11.79	3.62	48.03	23.05
				8	6.18	3.22	52.10	9.02
			Mean			4.06	45.09	76.87

## Data Availability

The data presented in this study are available on request from the corresponding author. The data are not publicly available due to project confidentiality.
